# C-reactive protein serum levels as an early predictor of outcome in patients with pandemic H1N1 influenza A virus infection

**DOI:** 10.1186/1471-2334-10-288

**Published:** 2010-10-04

**Authors:** Ofer Zimmerman, Ori Rogowski, Galit Aviram, Michal Mizrahi, David Zeltser, Dan Justo, Esther Dahan, Roy Arad, Oholi Touvia, Luba Tau, Jalal Tarabeia, Shlomo Berliner, Yael Paran

**Affiliations:** 1 Department of Internal Medicine D, Tel-Aviv Sourasky Medical Center and the Sackler Faculty of Medicine, Tel-Aviv University, Tel-Aviv, Israel; 2 Department of Radiology, Tel-Aviv Sourasky Medical Center and the Sackler Faculty of Medicine, Tel-Aviv University, Tel-Aviv, Israel; 3 Department of Internal and Geriatric Medicine B, Tel-Aviv Sourasky Medical Center and the Sackler Faculty of Medicine, Tel-Aviv University, Tel-Aviv, Israel; 4 Unit of Intensive Care, Tel-Aviv Sourasky Medical Center and the Sackler Faculty of Medicine, Tel-Aviv University, Tel-Aviv, Israel; 5 Epidemiology and Preventive Medicine Unit, Tel-Aviv Sourasky Medical Center and the Sackler Faculty of Medicine, Tel-Aviv University, Tel-Aviv, Israel; 6 Infectious Diseases Unit, Tel-Aviv Sourasky Medical Center and the Sackler Faculty of Medicine, Tel-Aviv University, Tel-Aviv, Israel

## Abstract

**Background:**

Data for predicting which patients with pandemic influenza A (H1N1) infection are likely to run a complicated course are sparse. We retrospectively studied whether the admission serum C-reactive protein (CRP) levels can serve as a predictor of illness severity.

**Methods:**

Included were all consecutive adult patients who presented to the emergency department (ED) between May-December, 2009 with a flu-like illness, a confirmed diagnosis of pandemic influenza A (H1N1) infection and a serum CRP level measured within 24 hours of presentation. Patients with a proven additional concurrent acute illness (e.g., bacteremia) were excluded. We used the ROC curve analysis, Kaplan-Meier curves and the Cox proportional hazard model to evaluate the predictive ability of CRP as a prognostic factor.

**Results:**

Seventeen (9%) of the 191 enrolled patients were admitted to the intensive care unit (ICU), of whom eight (4%) required mechanical ventilation and three (2%) died. The median admission serum CRP levels were significantly higher among patients who required subsequent ICU care and mechanical ventilation than among patients who did not (123 mg/L and 112 mg/L vs. 40 mg/L, *p *< .001 and 43 mg/L, *p *= .017, respectively). A Cox proportional hazard model identified admission serum CRP levels and auscultatory findings over the lungs as independent prognostic factors for ICU admission. Admission serum CRP levels were the only independent prognostic factor for mechanical ventilation. Thirty days after presenting to the ED, none of the patients with admission serum CRP level <28 mg/L (lower tertile) required either ICU admission or mechanical ventilation. At the same time point, 19% of the patients with admission serum CRP level ≥70 mg/L (upper tertile) needed to be admitted to the ICU and 8% of the same upper tertile group required mechanical ventilation. The differences in the rates between the lower vs. upper tertile groups were significant (Log-Rank *p *< .001 for ICU and *p *< .024 for mechanical ventilation).

**Conclusions:**

In our study group, serum CRP levels obtained in the early ED admission stage from patients presenting with pandemic H1N1 influenza A infection were found to serve as a useful gauge for predicting disease course and assisting in patient management.

## Background

The clinical manifestations of pandemic H1N1 influenza A infection range from a relatively mild and self-limiting respiratory infection to a severe illness with significant morbidity and mortality [1,2]. There is no single laboratory test that can serve as a potential biomarker to identify the patients at high risk for a complicated clinical course. Shortly after the pandemic reached our catchment area, we noted that some of the patients who needed to be hospitalized for severe H1N1 infection had high serum levels of C-reactive protein (CRP), which are usually seen in systemic bacterial infections and not in viral respiratory diseases [3-6]. High serum CRP levels were found in patients affected by Corona virus during the severe acute respiratory syndrome (SARS) outbreak in 2002 [7,8], when they were also identified as predictors of respiratory failure and death [7-9].

The current retrospective study examined serum CRP levels obtained during the first 24 hours since admission to emergency department (ED) as a predictor of illness severity among patients infected with the 2009 pandemic influenza A (H1N1). The evaluation compared the predictive capacity of serum CRP levels with that of other factors, such as underlying medical conditions, vital signs, physical signs, chest radiograph findings and laboratory test results.

## Methods

### Participants

The study was approved by the local ethics committee of the Tel Aviv Sourasky Medical Center, and informed patient consent was waived due to the observational nature of the study. The setting for this investigation was a single tertiary care university-affiliated medical center. The Tel Aviv Sourasky Medical Center serves a population of 1 million citizens in the Tel Aviv municipal area. All of the patients were 18 years of age or older. Data were collected for all consecutive patients admitted to the ED between May 1 and December 31, 2009, who fulfilled the clinical criteria for confirmed H1N1 influenza infection as established by the United States Centers for Disease Control and Prevention (CDC). These criteria included flu-like symptoms, such as a body temperature of 37.8°C (100°F) or higher, cough or sore throat, and a real-time reverse transcriptase polymerase chain reaction assay (RT-PCR) positive for H1N1 virus [10]. All patients whose serum CRP levels were measured within 24 hours of presentation to the ED were eligible for inclusion, whether they were subsequently hospitalized or discharged after initial evaluation. Following a review of patients' charts and records for symptoms and signs, laboratory and imaging findings, and blood and urine cultures (when available), patients with a proven additional concurrent acute illness (e.g., bacteremia) were excluded from the study.

### Study Design

Patients' records were reviewed for demographics, background diseases (including obesity, diabetes, current smoking, asthma, chronic obstructive pulmonary disease (COPD), ischemic heart disease (IHD), congestive heart failure (CHF), and active cancer), pregnancy, permanent steroid therapy, current chemotherapy, physical signs, vital signs, laboratory findings, chest radiograph findings, length of hospitalization, admission to the intensive care unit (ICU), need for mechanical ventilation, and death. The following times were also noted: from the first symptom to admission, from admission to ICU, provision of mechanical ventilation, and death. Radiologists unaware of patients' clinical data and outcome reviewed all chest radiographs obtained within 24 hours of admission to the ED, for findings characteristic of H1N1 pneumonia in adults, according to recently published descriptions [11,12]. The main outcome measures were whether or not the patient was admitted to the ICU, if mechanical ventilation was required and death. Follow-up of all the enrolled patients was managed via a telephone survey conducted at least 30 days since hospital admission. The query items focused on the appearance of any additional symptoms, the administration of any additional medical treatment, re-hospitalization, and outcome.

### Statistical Analysis

Values are presented as means ± SD or median and interquartile range (IQR) for the continuous variables and as number of individuals (and the percentage in each group) for the categorical variables. The comparison of continuous variables between groups was by independent samples Student's t-test for normally distributed variables and by the Mann-Whitney U statistics for non-normally distributed variables. Frequencies of categorical variables were compared by the Fisher exact test. In order to assess CRP as an early predictor of outcome, a receiver operating characteristic (ROC) curve was plotted and the area under the curve was calculated to determine the predictive ability of different CRP serum level cutoff values for the different outcomes [13]. Primary outcomes of required transfer to ICU and mechanical ventilation were evaluated on a time-to-event basis by Kaplan-Meier analysis with the use of the log-rank statistics to test for determining differences in the rates of the end points according to serum CRP levels. Cox proportional-hazard modeling was used, applying the forward stepwise analysis method to assess the effects of potential outcome markers. The SPSS statistical package was used to perform all the statistical evaluations (SSPS Inc., Chicago, IL, USA). For all analyses, a value of *p *< .05 (two-tailed) was considered to indicate statistical significance.

## Results

During the study period, 315 adult patients who fulfilled the CDC clinical criteria for confirmed H1N1 influenza virus infection [10] were admitted to the ED. In 209 of these patients (66%), serum CRP levels were measured within 24 hours of admission. Blood and urine cultures were obtained on admission in 110 (53%) and in 65 (31%) of these 209 patients, respectively. Eighteen patients (9%) were excluded from this study due to a concurrent acute illness on presentation. Consequently, the final study cohort comprised 191 patients whose serum CRP levels were obtained within 24 hours of admission and whose sole acute illness was a confirmed diagnosis of H1N1 influenza-virus infection. Patients' mean age was 43 ± 16 years (range 18-85). There were 95 (50%) female and 96 (50%) male patients. The most common background medical conditions were active smoking (n = 50, 26%), asthma (n = 37, 19%), diabetes mellitus (n = 24, 13%) and COPD (n = 22, 12%).

The median time from symptom onset until presentation to the ED was 72 hours (IQR 48-120). The median time from admission until first serum CRP level determination was 1 hour and 17 minutes. One hundred sixty eight (88%) patients were hospitalized and 23 (12%) were discharged to their homes after the initial evaluation in the ED. The median length of hospitalization was 3 days [2-5].

Seventeen (9%) patients were admitted to the ICU, of whom eight (4%) required mechanical ventilation and three died (2%). All 185 (98%) of the 188 surviving patients contacted through a follow-up telephone survey described an unremarkable clinical course of the disease in the thirty days since they presented at the ED.

With the exception of one patient who was transferred directly from the ED to the ICU, all of the admitted patients were first transferred from the ED to a hospital ward. Sixteen patients were subsequently transferred to the ICU. The median time from admission to the ED to transfer to the ICU was 53 hours (IQR 23-65). There was no significant difference in the prevalence of background medical conditions among the patients who required hospitalization in the ICU and those who did not (data not shown). The median time between the onset of first symptoms until presentation to the ED was similar for both hospitalized groups (Table 1).

**Table 1:** Comparison between variables of ICU and non-ICU patients

Variable	ICU (n = 17)	Non-ICU (n = 174)	*p *value
Time to ED (hours), median (IQR)*	96 (24 - 168)	72 (48 - 120)	0.506
Sys BP (mmHg), median (IQR)	128 (104 - 148)	125 (114 - 140)	0.904
Hypotension (sys BP < 90 mmHg), n (%)	0 (0)	4 (2.5)	> 0.999
Dias BP (mmHg), median (IQR)	80(62 - 95)	77 (69 - 84)	0.619
Heart rate (beats/min), median (IQR)	100 (81 - 118)	96 (86 - 108)	0.640
Tachycardia (> 100 beats/min), n (%)	8 (47)	61 (38)	0.601
Fever (°C), median (IQR)	37.9 (37.2 - 38.3)	37.5 (37.1 - 38.3)	0.578
High fever (≥39.0°C), n (%)	2 (12)	25 (15)	0.751
Saturation (%), median (IQR)	92 (87 - 96)	97 (94 - 98)	0.006
Low saturation (≤90%), n (%)	7 (44)	18 (12)	0.003
Throat redness, n (%)	2 (12)	35 (20)	0.534
Lung auscultatory findings, n (%)	12 (71)	54 (31)	0.002
Dyspnea, n (%)	6 (35)	39 (22)	0.369
Stupor, n (%)	1 (6)	2 (1.1)	0.245
Pneumonia on chest X-ray, n (%)	12 (71)	62 (39)	0.018
WBC (×10^3^/ml^3^), median (IQR)	7.4 (4.9 - 9.3)	7.8 (5.6 - 10.7)	0.611
% PNM (%), median (IQR)	85 (78 - 89)	78 (70 - 85)	0.010
PMN (×10^3^/ml^3^), median(IQR)	5.9 (4.2 - 8.1)	5.6 (3.9 - 8.7)	0.750
% LYM (%), median(IQR)	8 (6 - 14)	12 (7 - 19)	0.087
LYM (×10^3^/ml^3^), median(IQR)	0.6 (0.4 - 1.1)	0.9 (0.6 - 1.3)	0.057
Platelets (×10^3^/ml^3^), median (IQR)	203 (155 - 222)	209 (169 - 251)	0.307
Creatinine (mg/dl), median (IQR)	1.1 (0.9 - 1.4)	1.1 (0.9 - 1.3)	0.603
Bilirubin (mg/dl), median (IQR)	0.5 (0.3 - 0.7)	0.5 (0.3 - 0.7)	0.882
Alkaline phosphatase (U/l), median (IQR)	62 (47 - 91)	55 (4 - 76)	0.347
ALT (mg/dl), median (IQR)	28 (18 - 47)	25 (17 - 34)	0.373
GGT (mg/dl), median (IQR)	29 (10 - 47)	30 (15 - 39)	0.863
CRP (mg/L), median (IQR)	123 (69 - 184)	40 (20 - 82)	< 0.001

*Time from symptom onset until arrival to the ED

*ED*, emergency department; *ICU*, intensive care unit; *Sys*, systolic; *Dias*, diastolic; *IQR*, interquartile range; *WBC*, white blood cells; *PMN*, polymorphonuclears; *Lym*, lymphocytes; *ALT*, alanine aminotransferase; *GGT*, gamma-glutamyl transpeptidase; *CRP*, C-reactive protein

Low blood oxygen saturation (***≤ ***90%) on presentation to the ED and abnormal auscultatory findings over the lung fields (bronchial breath sounds or crackles) on admission were more common in the patients who were eventually admitted to ICU compared to those who remained in the wards (Table 1). The same clinical observation was noted in regard to patients who required mechanical ventilation compared to those who did not; however, in this comparison, the difference was not statistically significant (data not shown). Seventy-four of the 177 (93%) patients who underwent chest radiography within 24 hours of admission had findings consistent with pneumonia. The prevalence of these findings was significantly higher among the patients who required ICU care compared with those who did not (Table 1). Chest radiographic findings consistent with pneumonia were also more prevalent among patients who required mechanical ventilation than among those who did not, but this difference was not significant (6/8 (75%) vs. 68/170 (40%), respectively, *p *= .069).

Among the evaluated laboratory parameters, serum CRP levels and neutrophil percentage in a complete blood count (CBC) obtained within 24 hours of admission comprised the only two factors that significantly differentiated between patients who needed to be admitted to the ICU and those who could remain on the ward (Table 1). Unfortunately, we could not include the parameter of patients' blood gases in the current analysis, since such a sample was obtained from only a few of the patients.

**Figure 1:**
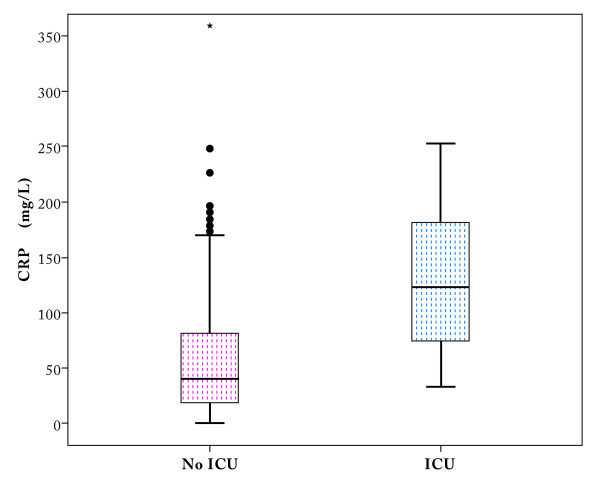
**Box-plot of serum C-reactive protein levels on admission in patients who required admission to an intensive care unit compared to those who did not**. The *dotted boxes *represent the interquartile range (25th to 75th percentiles), the *thick black line *in the box is the 50th percentile (median), and the *bars *represent the range of results, excluding outliers. The *black dots *are "outliers" and the *black star* is an "extreme outlier".

**Figure 2:**
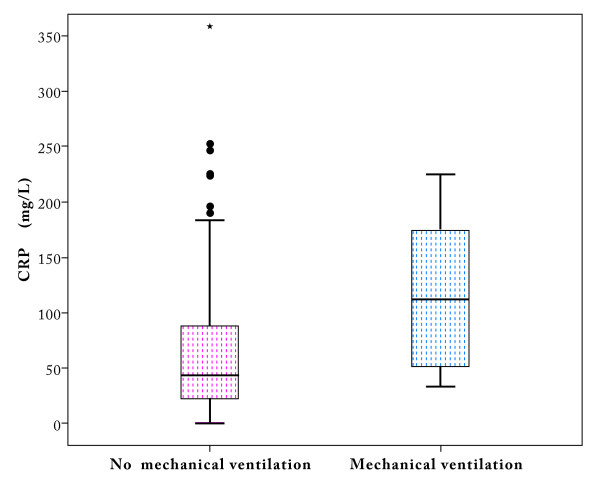
**Box-plot of serum C-reactive protein levels on admission in patients who required mechanical ventilation compared to those who did not**. The *dotted boxes *represent the interquartile range (25th to 75th percentiles), the *thick black line *in the box is the 50th percentile (median), and the *bars *represent the range of results, excluding outliers. The *black dots *are "outliers" and the *black star* is an "extreme outlier".

Serum CRP levels were the only laboratory parameter that significantly differentiated between patients who required mechanical ventilation and those who did not (112 mg/L [IQR 45-180] vs. 43 mg/L [IQR 22-89], respectively, *p *= .017). Figures 1 and 2 display box-plots of serum CRP levels, according to ICU admission and need for mechanical ventilation, respectively.

The ability to consider serum CRP levels obtained during the first 24 hours of ED admission as a biomarker for predicting pandemic H1N1 influenza A patients' need for ICU care or mechanical ventilation was evaluated using ROC curve analysis. The small number of deaths in our cohort precluded the performing of ROC curve analysis for that outcome. The area under the curve (AUC) for ICU admission was 0.82 (95% CI = 0.73-0.91; *p *< .001). The AUC for mechanical ventilation was 0.75 (95% CI = 0.60-0.90; *p *= .017). The sensitivity, specificity and positive and negative predictive values (PPV and NPV) for ICU admission and mechanical ventilation of serum CRP at different cut-off levels are displayed in Table 2.

**Table 2:** ROC curve analysis describing the sensitivity, specificity, negative-and positive-predictive values for ICU admission and mechanical ventilation at different cut-off levels.

ICU				
**Serum CRP (mg/L) cut-off level**	**Sensitivity**	**Specificity**	**PPV**	**NPV**

33	100	42	14	100
39	94	49	15	99
60	88	63	19	98
64	82	68	20	98
75	77	72	21	97
98	71	81	27	97
112	59	83	25	95
123	53	86	27	95
149	47	90	32	95

**Mechanical ventilation**				

**Serum CRP (mg/L) cut-off level**	**Sensitivity**	**Specificity**	**PPV**	**NPV**

33	100	40	7	100
39	88	46	7	99
64	75	65	9	98
102	63	79	11	98
123	50	84	12	98
164	38	91	16	97

*PPV*, positive predictive value, *NPV*, negative predictive value

The Cox proportional hazard model was applied to assess the ability of the following factors to predict the need for ICU admission: low oxygen saturation (***≤***90%), abnormal auscultatory findings over lung fields, chest radiograph findings consistent with pneumonia, percentage of neutrophil in a CBC, and serum CRP levels. The first variable that entered the model was CRP (*p *= .001), followed by abnormal auscultatory findings (*p *= .028). None of the other three variables entered the model afterwards. The CRP hazard ratio (95% confidence interval [CI]) for transfer to the ICU was 1.09 (1.06-1.12) for each increase of 10 mg/L in CRP level. The hazard ratio (95% CI) of presence of auscultatory findings over the lung fields for being transferred to the ICU was 3.85 (1.16-12.8).

The Cox proportional hazard model was applied to assess the ability of serum CRP level to predict the need for mechanical ventilation. The CRP level was the only variable that entered the model (*p *= .018), and the hazard ratio (95% CI) for mechanical ventilation was 1.09 (1.05 - 1.13) for each increase of 10 mg/L in CRP level.

**Figure 3:**
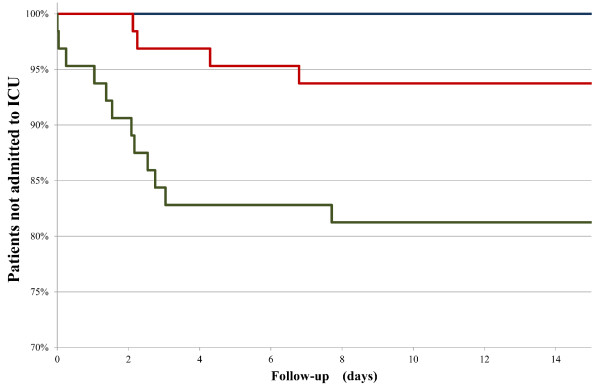
**Kaplan Meier analysis displaying the cumulative probability of not reaching the outcome measure of intensive care unit admission for patients according to tertiles of serum CRP levels on admission to the emergency department**. The *blue line *represents <28 mg/L, the *red line *28-69 mg/L, and the *green line *≥70 mg/L.

**Figure 4:**
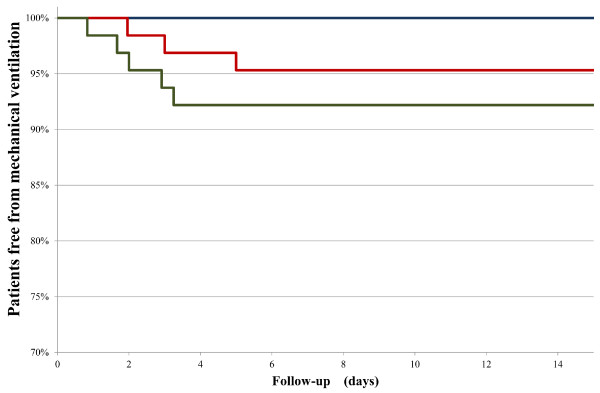
**Kaplan Meier analysis displaying the cumulative probability of not reaching the outcome measure of mechanical ventilation for patients according to tertiles of serum CRP levels on admission to the emergency department**. The *blue line *represents <28 mg/L, the *red line *28-69 mg/L, and the *green line *≥70 mg/L.

A Kaplan-Meier estimate of the two main outcome measures, i.e., the need for ICU admission and for mechanical ventilation, was performed using tertiles of serum CRP levels (i.e., <28, 28-69, and ≥70 mg/L). At the 30-day time point since their presentation to the ED, none of the patients with a serum CRP level <28 mg/L (lower tertile) needed to be admitted to the ICU or required mechanical ventilation (Figures 3 and 4). At the same time point, 19% of the patients with a serum CRP level ≥70 mg/L (upper tertile) needed to be admitted to the ICU and 8% required mechanical ventilation. The differences in the rates between the lower vs. upper tertile groups were significant (Log-Rank *p *< .001 for ICU and *p *< .024 for mechanical ventilation).

## Discussion

The results of the study confirm that low saturation (< 90%), auscultatory findings over the lung fields, findings consistent with pneumonia on chest radiography, high percentage of neutrophil in a CBC and high serum CRP levels at the time of presentation to the ED are associated with a severe course of H1N1 influenza A virus infection. Of these variables, serum CRP levels were found to be the best predictor of subsequent need for ICU admission and the only predictor of subsequent need for mechanical ventilation.

Moreover, low initial serum CRP levels were an excellent predictor of good outcome: at 30 days from admission, none of the patients with CRP levels lower than 33 mg/L required either ICU care or mechanical ventilation. The fact that patients with low serum CRP levels enjoyed good outcome may serve as a guiding tool, enabling the assessing clinician in the ED to differentiate between "low risk" patients vs. "high risk" patients. As evident from Table 2, a cut-off value of 33 mg/L is a viable choice that achieves 100% sensitivity but a specificity of only around 40% for ICU admission or mechanical ventilation. By comparison, a slightly higher CRP cut-off of 64 mg/L achieved lower but acceptable sensitivities of 82% and 75% for ICU admission and mechanical ventilation, respectively, with higher specificities of 68% and 65%, respectively. Thus, it appears that serum CRP levels may have a significant role in helping the physician in the decision making process on the individual patient level.

The high serum CRP levels that were observed among some of the patients in our series are usually found among patients with bacterial infections [3-6]. Two series of studies of lung specimens taken from autopsies of patients who died in the recent pandemic reported that many of the victims had a proven secondary bacterial infection. Gill et al. [14] found histologic and microbiologic evidence of bacterial pneumonia in 55% of 34 people who died following a confirmed pandemic influenza A H1N1 virus infection. Similar findings were found in 29% of lung specimens from 77 fatal cases in another series [15]. Thus, it is plausible that the high CRP levels among some of the patients in the current study may represent a secondary bacterial infection. Another possible explanation is that the high serum CRP levels represent a severe inflammatory response to the H1N1 virus. A similar hypothesis was suggested with regards to SARS, which was caused by the Corona virus in the 2002 outbreak. It was postulated that exaggerated activation of cytokines/chemokines played an significant role in the pathogenesis of the disease leading to an adverse outcome [16,17]. Either one of the aforementioned explanations is compatible with the current findings, which indicate that CRP levels may serve as a useful biomarker for predicting disease severity among patients with suspected or confirmed H1N1 influenza A virus infection.

One of the major limitations of the current work is its retrospective nature and the relatively small number of patients with a severe outcome. The absence of an agreed protocol for the measurement of serum CRP in patients with H1N1 virus infection admitted to the ED led to the exclusion of 34% of the 315 patients from the study cohort and may have also biased the results. Thus, it may make it difficult to extrapolate the results to another population. Despite these limitations, the association between CRP levels upon presentation to the ED and patient outcome was significant and substantial. Another potential limitation is the need for ICU care as a measurement of outcome severity, since the decision itself might have been based on, among other factors, admission serum CRP levels. Only one patient in this series was admitted to the ICU directly from the ED.

## Conclusions

In the current sample of patients with pandemic H1N1 influenza A infection, the serum CRP levels obtained during the first 24 hours since presentation to the ED were found to be significantly correlated with impending disease severity. Based on this finding, it is plausible that this measurement may serve as a useful early biomarker to identify patients at high risk for a complicated clinical course of the disease. The ability to differentiate between high and low risk patients has clear-cut clinical and therapeutic implications for decision-making on issues of patient management.

## Abbreviations

CRP: C-reactive protein; ED: Emergency department; ICU:Intensive care unit; SARS: Severe acute respiratory syndrome; CDC: Centers for Disease Control and Prevention; RT-PCR: Real-time reverse transcriptase polymerase chain reaction assay; COPD: Chronic obstructive pulmonary disease; IHD: Ischemic heart disease; CHF: Congestive heart failure; IQR: Interquartile range; ROC: Receiver operating characteristic; CBC: Complete blood count; AUC: Area under the curve; PPV: Positive predictive values; NPV: Negative predictive values; SYS: Systolic; DIAS: Diastolic; WBC: White blood cells; PMN: Polymorphonuclears; LYM: Lymphocytes; ALT: Alanine aminotransferase; GGT: Gamma-glutamyl transpeptidase

## Competing interests

The authors declare that they have no competing interests.

## Authors' contributions

OZ participated in the conception, design and coordination of the study, acquisition of data, interpretation of data and manuscript drafting; OR performed the statistical analysis and participated in the design of the study, interpretation of data and manuscript drafting; GA carried out the interpretation of chest radiographs and revised the manuscript for important intellectual content; MM participated in the coordination of the study, acquisition of data and revised the manuscript for important intellectual content; DZ participated in the conception and design of the study and revised the manuscript for important intellectual content; DJ participated in the interpretation of data and took part in the manuscript drafting; ED participated in the interpretation of the data and revised the manuscript for important intellectual content; RA took part in the study design and acquisition of data and revised the manuscript for important intellectual content; OT took part in the study design and acquisition of data and revised the manuscript for important intellectual content; LT took part in the study design and acquisition of data and revised the manuscript for important intellectual content; JT took part in the acquisition of data and revised the manuscript for important intellectual content; SB participated in the study design, interpretation of data and manuscript drafting; YP participated in the conception, design and coordination of the study, acquisition of data, interpretation of data and manuscript drafting. All authors read and approved the final manuscript.

## Pre-publication history

The pre-publication history for this paper can be accessed here:

http://www.biomedcentral.com/1471-2334/10/288/prepub
